# Targeting of nicotinamide phosphoribosyltransferase enzymatic activity ameliorates lung damage induced by ischemia/reperfusion in rats

**DOI:** 10.1186/s12931-017-0557-2

**Published:** 2017-04-24

**Authors:** Geng-Chin Wu, Wen-I Liao, Shu-Yu Wu, Hsin-Ping Pao, Shih-En Tang, Min-Hui Li, Kun-Lun Huang, Shi-Jye Chu

**Affiliations:** 1 The Graduate Institute of Medical Sciences, National Defense Medical Center, Taipei, Taiwan; 2 Department of Internal Medicine, Taoyuan Armed Forces General Hospital, Taoyuan, Taiwan; 3 Department of Emergency Medicine, Tri-Service General Hospital, Taipei, Taiwan; 4 The Graduate Institute of Aerospace and Undersea Medicine, National Defense Medical Center, Taipei, Taiwan; 5 Division of Pulmonary and Critical Care Medicine, Department of Internal Medicine, Tri-Service General Hospital, Taipei, Taiwan; 6 Department of Physical Medicine and Rehabilitation, Kaohsiung Veterans General Hospital, Kaohsiung, Taiwan; 7 Department of Internal Medicine, Tri-Service General Hospital, National Defense Medical Center, No. 325, Section 2, Chenggong Road, Neihu 114, Taipei, Taiwan

**Keywords:** Acute lung injury, Ischemia-reperfusion, Visfatin, Nicotinamide phosphoribosyltransferase, pre-B cell colony-enhancing factor

## Abstract

**Background:**

Emerging evidence reveals that nicotinamide phosphoribosyltransferase (NAMPT) has a significant role in the pathophysiology of the inflammatory process. NAMPT inhibition has a beneficial effect in the treatment of a variety of inflammatory diseases. However, it remains unclear whether NAMPT inhibition has an impact on ischemia-reperfusion (I/R)-induced acute lung injury. In this study, we examined whether NAMPT inhibition provided protection against I/R lung injury in rats.

**Methods:**

Isolated perfused rat lungs were subjected to 40 min of ischemia followed by 60 min of reperfusion. The rats were randomly allotted to the control, control + FK866 (NAMPT inhibitor, 10 mg/kg), I/R, or I/R + FK866 groups (*n* = 6 per group). The effects of FK866 on human alveolar epithelial cells exposed to hypoxia-reoxygenation (H/R) were also investigated.

**Results:**

Treatment with FK866 significantly attenuated the increases in lung edema, pulmonary arterial pressure, lung injury scores, and TNF-α, CINC-1, and IL-6 concentrations in bronchoalveolar lavage fluid in the I/R group. Malondialdehyde levels, carbonyl contents and MPO-positive cells in lung tissue were also significantly reduced by FK866. Additionally, FK866 mitigated I/R-stimulated degradation of IκB-α, nuclear translocation of NF-κB, Akt phosphorylation, activation of mitogen-activated protein kinase, and downregulated MKP-1 activity in the injured lung tissue. Furthermore, FK866 increased Bcl-2 and decreased caspase-3 activity in the I/R rat lungs. Comparably, the in vitro experiments showed that FK866 also inhibited IL-8 production and NF-κB activation in human alveolar epithelial cells exposed to H/R.

**Conclusions:**

Our findings suggest that NAMPT inhibition may be a novel therapeutic approach for I/R-induced lung injury. The protective effects involve the suppression of multiple signal pathways.

## Background

Intracellular nicotinamide phosphoribosyltransferase (NAMPT), also called visfatin or pre-B cell colony-enhancing factor, is the rate-limiting enzyme in the salvage pathway for nicotinamide adenine dinucleotide (NAD) biosynthesis. It influences the function of NAD-dependent enzymes such as sirtuins and poly (ADP-ribose) polymerases, which regulate cellular signaling, insulin resistance, apoptosis, oxidative stress response, and inflammation [1]. All tissues and cells contain NAMPT. The ubiquitous expression of NAMPT implies the pleiotropic actions of proteins in cellular events [1]. Moreover, NAMPT can also be found as an extracellular secreted form under inflammatory conditions; it is a potent extracellular proinflammatory inducer of the NF-kB pathway, toll-like receptor signaling, apoptosis, and leukocyte extravasation signaling [2]. Because of the upregulation of NAMPT expression in a variety of inflammatory responses, it is implicated in the pathogenesis of various inflammatory disorders, such as atherosclerosis, psoriasis, inflammatory bowel disease, rheumatoid arthritis, and myocardial failure [1]. Recent reports indicate that a low molecular-weight inhibitor of NAMPT, FK866, improves inflammation-related diseases in an animal model, including spinal cord injury, lipopolysaccharide (LPS)-induced myocardial impairment, myocardial infarction, inflammatory arthritis, endotoxic shock, and autoimmune encephalitis [3–6]. These findings suggest that NAMPT could be a novel therapeutic target in various disorders, but a better understanding of its mechanisms of action is a prerequisite for the use of a NAMPT inhibitor as a therapeutic option in relevant diseases.

Despite advances in research in acute lung injury/acute respiratory distress syndrome (ALI/ARDS), the fundamental basis for ischemia-reperfusion (I/R)-evoked pathophysiology remains unclear. Recently, NAMPT was demonstrated as a potential novel biomarker in ALI/ARDS via genomic and genetic studies [7, 8]. The expression of NAMPT in the lungs is markedly increased in human and animal models of ALI, and NAMPT levels are significantly increased in serum and bronchoalveolar lavage fluid (BALF) [7]. Variations in *NAMPT* polymorphisms were also significantly associated with susceptibility to sepsis and ALI [7, 8]. Furthermore, heterozygous *NAMPT* ^+/-^ mice were significantly protected from the development and severity of ventilator-induced lung injury (VILI) [9]. Moreno-Vinasco et al. recently reported that FK866, an inhibitor of NAMPT enzymatic function, had beneficial effects in VILI and LPS-induced lung injury [10]. Matsuda et al. demonstrated that FK866 protected against intestinal I/R-associated ALI in mice. The protective effect of FK866 occurred via modulation of the NF-*κ*B pathway [11]. These investigations strongly support a potentially important role for NAMPT in the inflammatory processes observed in ALI/ARDS.

I/R in the lungs can lead to ALI that was obviously different from the study of Matsuda et al. that ALI was associated with I/R at distant, nonpulmonary sites [11]. I/R-induced ALI is the major cause of primary graft dysfunction in the early stages after lung transplantation. The morbidity and mortality associated with I/R-induced ALI is still high [12]. Therefore, it is important to explore the molecular mechanisms of I/R-induced ALI and develop an effective therapy. In this study, we further determined the role of NAMPT enzymatic activity in the pathogenesis of I/R-induced acute lung injury using an inhibitor of NAMPT enzymatic function, FK-866.

## Methods

### Isolated perfused rat lung model

Care of the rats used in this experiment met the guidelines set forth by the National Institutes of Health (National Academy Press, 1996). The Animal Review Committee of National Defense Medical Center approved the study protocol. Rat lungs were isolated and perfused as previously described [13–15]. Briefly, Sprague-Dawley male rats (350 ± 20 g) were ventilated with humidified air containing 5% CO_2_ at a tidal volume of 3 ml, a positive end-expiratory pressure of 1 cm H_2_O, and a rate of 60 breaths/min. After a sternotomy, heparin (1 U/g of body weight, [BW]) was injected into the right ventricle, and 10 mL of intracardiac blood was withdrawn. The pulmonary artery and the left ventricle were cannulated and perfused with a physiological salt solution (119 mM NaCl, 4.7 mM KCl, 1.17 mM MgSO_4_, 22.6 mM NaHCO_3_, 1.18 mM KH_2_PO_4_, 1.6 mM CaCl_2_, 5.5 mM glucose, and 50 mM sucrose) containing 4% bovine serum albumin. The 10 ml of collected blood was added to the perfusate as a “half-blood” solution before recirculation. The constant flow rate of the roller pump was maintained at 8–10 ml/min. The recirculating perfusate with the isolated lungs *in situ* was placed on an electronic balance to record real-time changes in lung weight (LW). The left atrial pressure, representing the pulmonary venous pressure (PVP), and the pulmonary arterial pressure (PAP) were continuously monitored from the side arm of the cannula.

### Vascular filtration coefficient

The vascular filtration coefficient (K_f_) was calculated from the change in lung weight caused by elevation of venous pressure as described previously [14–16]. K_f_ was defined as the y-intercept of the plot (g min^−1^) divided by the PVP (10 cmH_2_O) and lung weight, and expressed in whole units of g · min^−1^ · cmH_2_O^−1^ × 100 g [14–16].

### Lung weight/body weight and wet/dry (W/D) weight ratios

The right lung was removed after the experiments in the hilar region. The wet lung weight was then determined, and the LW/BW ratio was calculated. For the dry weight, a part of the right upper lung lobe was dried for 48 h at 60 °C in an oven, and the W/D weight ratio was calculated.

### Assessment of total cell counts, BALF protein, cytokine-induced neutrophil chemoattractant-1(CINC-1), interleukin-6 (IL-6), and tumor necrosis factor-α (TNF-α) levels

BALF was obtained by lavaging the left lung twice with 2.5 ml of saline after the experiment. The lavage fluid was centrifuged at 200 × g for 10 min. The protein concentration in the supernatant was determined using a bicinchoninic acid protein assay kit (Pierce, Rockford, IL, USA). The levels of TNF-α, IL-6 and cytokine-induced neutrophil chemoattractant (CINC)-1 in the BALF were measured using a commercial ELISA kit (R&D Systems Inc., Minneapolis, MN, USA). Total cell counts in the BALF were assessed as described previously [15].

### Protein carbonyl contents and malondialdehyde levels in lung tissue

The lung tissue was homogenized in a 1.15% KCl aqueous solution. A 100-μL aliquot of the homogenized lung tissue was mixed into a solution of 200 μL of 8.1% thiobarbituric acid and 700 μL of distilled water. The mixture was then boiled for 30 min at 100 °C and centrifuged at 3000 × *g* for 10 min. The malondialdehyde content of the supernatant was measured by absorbance at 532 nm and was expressed as nmol/mg protein. The oxidative damage to the proteins in the lung tissue was assessed by determining the carbonyl group content based on a reaction with dinitrophenylhidrazine as previously described [15]. The carbonyl content was determined from the absorbance at 370 nm assuming a molar absorption coefficient of 220,000 M^−1^ and was expressed as the concentration of carbonyl derivatives in the protein (nmol carbonyl/mg protein) [15].

### Western blotting

Lung and cell culture protein lysates (30 μg/lane) were separated by 10–12% sodium dodecyl sulfate-polyacrylamide gel electrophoresis and immunoblots were developed as previously described [14, 15]. The blots were probed with primary antibodies against NAMPT (1:2000, Thermo Fisher Scientific, Rockford, IL, USA), B-cell lymphoma (Bcl)-2 (1:200, Santa Cruz Biotechnology, Dallas, Texas, USA), NF-κB p65, phospho-NF-κB p65, inhibitor of NF-κB (IκB)-α, extracellular signal-related protein kinase 1/2 (ERK1/2), phosho-ERK1/2, c-Jun N-terminal kinase (JNK), phospho-JNK, p38 protein kinase (p38), phospho-p38, and mitogen-activated protein kinase phosphatase-1 (MKP-1) (1:1000, Cell Signaling Technology, Danvers, MA, USA), proliferating cell nuclear antigen (PCNA) (1:1000, Abcam, Cambridge, MA, USA), and β-actin (1:10000, Sigma Chemical Company, St. Louis, MO, USA). The data are presented as the relative ratio of the target protein to the reference protein.

### Immunohistochemical analyses

Immunohistochemical staining to identify myeloperoxidase (MPO) and caspase-3 was performed as described previously [15, 17]. Briefly, paraffin-embedded lung tissue sections were deparaffinized before antigen retrieval. The slides were immersed in 3% H_2_O_2_ and 100% methanol for 15 min to quench endogenous peroxidase. Immunostaining of lung sections was done using a rabbit polyclonal antibody to MPO (1:100, Cell Signaling Technology) and the large activated fragment (17/19 kD) of caspase-3 (1:200; Cell Signaling Technology). The slides were washed and then incubated with rat-specific horseradish peroxidase polymer anti-rabbit antibody (Nichirei Corporation, Tokyo, Japan) for 30 min. Then, horseradish peroxidase substrate was added and reacted for 3 min, and the sections were counterstained with hematoxylin.

### Histopathology

The lung tissue was histologically prepared and stained with hematoxylin and eosin. The numbers of polymorphonuclear neutrophils in the interstitium were counted in 10 high-power fields (×400) and averaged. Two pathologists examined a minimum of 10 randomly selected fields in a masked fashion. Semiquantitative grading of lung injury on hematoxylin and eosin sections was done as previously described [15].

### Experimental design

The rat lungs were randomly assigned to receive normal saline (control, *n* = 6), FK866 (10 mg/kg, drug control, *n* = 6), I/R (*n* = 6), or I/R with FK866 (10 mg/kg, *n* = 6). FK866 (Alexis Biochemicals, San Diego, CA, USA) was added to the reservoir (containing 20 mL of perfusate). The doses of FK866 in this study were chosen according to previous investigations [4, 11]. The isolated lungs were allowed to equilibrate for 20 min before starting. The baseline PAP, PVP, weight change, and the initial *K*_f_ for 7 min were then measured. All parameters were equilibrated to baseline for 10 min after the measurements. In the I/R group, the lung preparations were kept at 25 °C. After all of the parameters had returned to the baseline state, the lungs were deflated by stopping ventilation and perfusion to cause ischemia. They were maintained in the deflated state for 40 min. Perfusion and ventilation were resumed, and the K_f_ was measured 60 min later.

### Hypoxia-reoxygenation (H/R) of A549 cells

Human type II alveolar epithelial cells (A549) were obtained from the Food Industry Research and Development Institute (BCRC 60074, Hsinchu, Taiwan) and maintained in F-12 K medium (Hyclone, Logan, UT, USA) containing 10% fetal bovine serum (Hyclone), penicillin, and streptomycin in a humidified atmosphere of 5% CO_2_-95% air. A549 cells were subjected to 24 h of hypoxia (1% O_2_-5% CO_2_-94% N_2_) followed by 4 h of reoxygenation (5% CO_2_-95% air) at 37 °C [14]. The cells were pretreated with vehicle or FK866 (50 nM) [4, 11]. The control group was continued in the reoxygenated state without the hypoxic stimulus. The supernatant was collected and assayed for IL-8 using a human IL-8 ELISA kit (R&D, Inc., Minneapolis, MN, USA).

### Data analysis

The data analysis was performed using GraphPad Prism 5 statistical software (GraphPad Software, San Diego, CA, USA). Data are expressed as means ± SD. The comparisons among the groups were conducted using one-way ANOVA followed by a *post-hoc* Bonferroni test. Two-way ANOVA for repeated measurements followed by the *post-hoc* Bonferroni test was using for comparisons of lung weight gain and PAP between groups. The significance level was defined as *P* < 0.05.

## Results

### Effect of FK866 on indices of lung edema

**Fig. 1:**
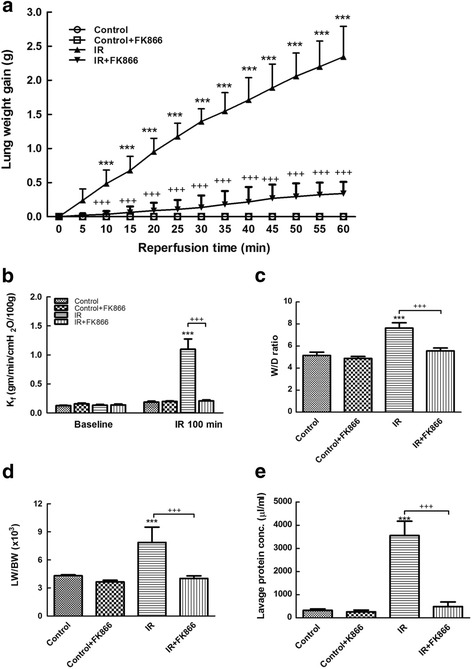
Effect of FK866 on pulmonary edema. Lung weight gain (**a**), K_f_ (**b**), lung wet/dry (W/D) weight ratios (**c**), lung weight/body weight (LW/BW) ratios (**d**), and protein concentrations in the bronchoalveolar lavage fluid (BALF) (**e**) significantly increased in the ischemia-reperfusion (IR) group. Treatment with FK866 significantly attenuated the increase in these parameters. Data are expressed as mean ± SD (*n* = 6 per group). ***p < 0.001, compared with the control group; ^+++^ *p* < 0.001, compared with the IR group

I/R significantly raised lung weight gain (Fig. 1a). The FK866 treatment reduced this increase in the lung weight gain. I/R significantly increased K_f_, LW/BW and W/D weight ratios, and protein concentrations in the BALF (*p <* 0.05*,* Fig. 1b–e); FK866 treatment significantly mitigated these increases.

### Effect of FK866 on PAP

**Fig. 2:**
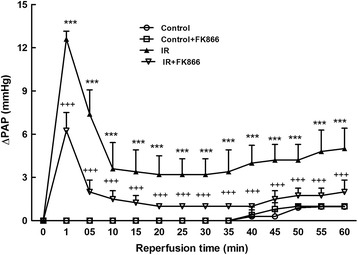
Effect of FK866 on pulmonary artery pressure (ΔPAP). PAP increased significantly in the ischemia-reperfusion (IR) group. The increase in PAP was attenuated significantly by treatment with FK866. Data are expressed as mean ± SD (*n* = 6 per group). ***p < 0.001, compared with the control group; ^+++^ *p* < 0.001, compared with the IR group

In the control group, the PAP remained steady during the 100-min observation period. In the I/R group, the PAP initially rose and then declined after reperfusion. After 60 min of reperfusion, the PAP in the I/R group was significantly higher than at baseline and that of the control group. Treatment with FK866 significantly diminished the increase of PAP in the I/R group (*p* < 0.05; Fig. 2).

### Effect of FK866 on NAMPT protein expression in lung tissue

**Fig. 3:**
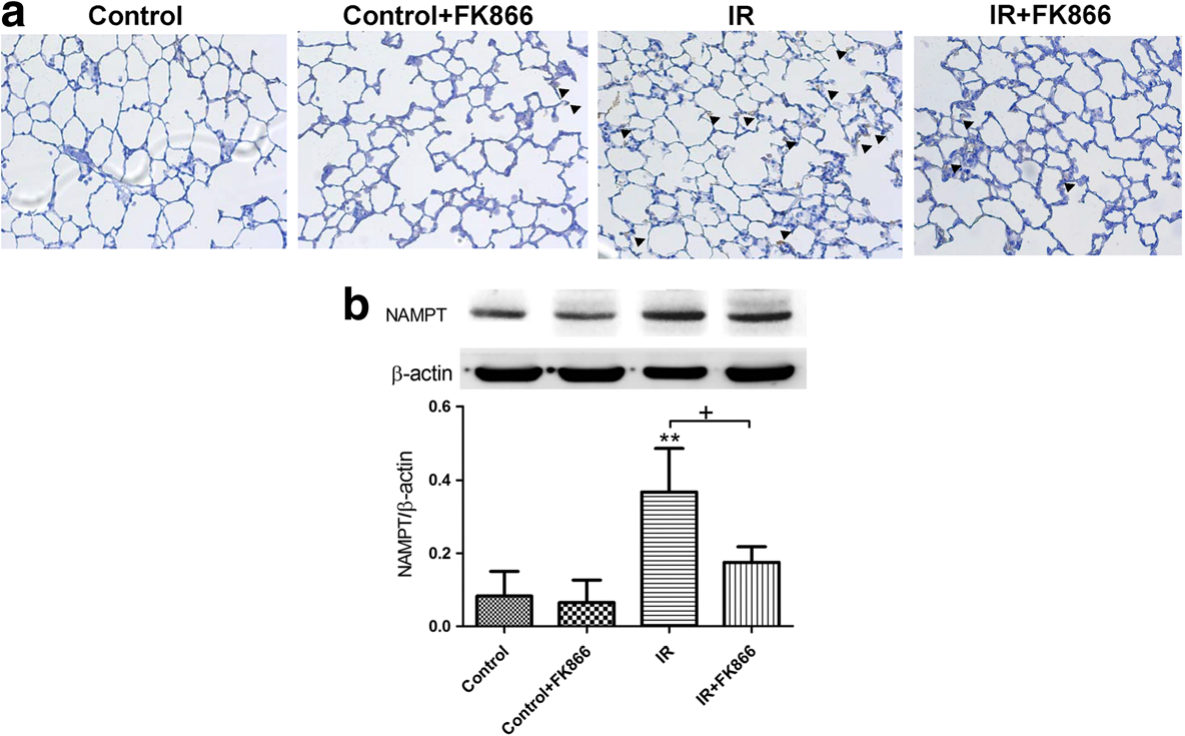
Effect of FK866 on NAMPT protein expression in lung tissue. **a** Immunohistochemistry for NAMPT in the lung (indicated with arrowhead) (200× magnification). **b** Western blot and densitometry analysis of NAMPT protein in the lung tissue. β-actin served as loading control for cytoplasmic proteins. Representative blots are shown. Ischemia-reperfusion (IR) significantly increased positive staining and protein expression of NAMPT in the lung tissue. FK866 significantly decreased the degree of NAMPT positive staining and protein expression. Data are expressed as mean ± SD (*n* = 6 per group). **p < 0.01, compared with the control group; ^+^ *p* < 0.05, compared with the IR group

There was faint or weak immunostaining of NAMPT in the lung sections of the control animals. In contrast, lung tissues had strong NAMPT staining after I/R injury. FK866 treatment reduced the intensity of staining in the lungs (Fig. 3a). Furthermore, I/R significantly increased NAMPT protein expression compared with that of the control group (*p* < 0.05; Fig. 3b). FK866 treatment significantly decreased NAMPT protein expression in the I/R group.

### Effect of FK866 on CINC-1, TNF-α, and IL-6 concentrations, and total cell counts in the BALF

**Fig. 4:**
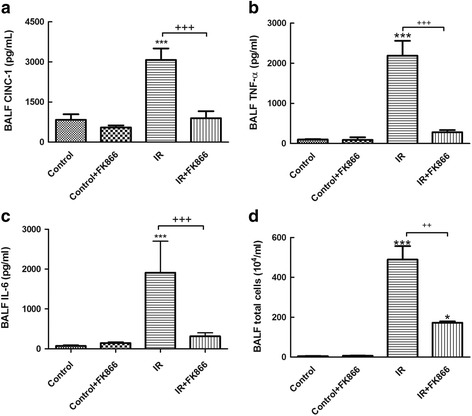
Effect of FK866 on CINC-1, TNF-α, and IL-6 levels, and total cell counts in bronchoalveolar lavage fluid (BALF). CINC-1 (**a**), TNF-α (**b**) and IL-6 (**c**) levels, and total cell counts (**d**) in the BALF significantly increased in the ischemia-reperfusion (IR) group. Treatment with FK866 significantly attenuated these increases in the BALF. Data are expressed as mean ± SD (*n* = 6 per group). ***p < 0.001, compared with the control group; ^+++^ *p* < 0.001, compared with the IR group

The concentrations of TNF-α, CINC-1 and IL-6, and total cell counts were significantly increased in the BALF of the I/R group compared with that of control group (*p* < 0.05; Fig. 4). FK866 significantly inhibited the I/R-mediated increases of TNF-α, CINC-1, IL-6 and total cell counts in the BALF (*p* < 0.05; Fig. 4).

### Effect of FK866 on carbonyl content, malondialdehyde level, and MPO-positive cells in lung tissue

**Fig. 5:**
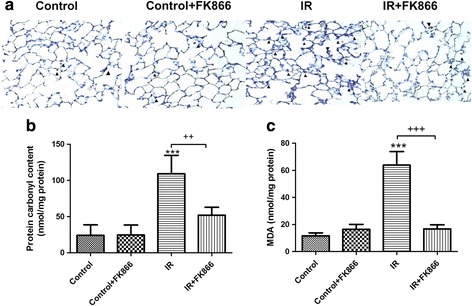
Effect of FK866 on protein carbonyl contents, MDA levels, and MPO-positive cells in lung tissue. MPO-positive cells (**a**), carbonyl contents (**b**), and MDA levels (**c**) in lung tissue significantly increased in the ischemia-reperfusion (IR) group. FK866 treatment significantly attenuated these increases. **a** Immunohistochemistry for MPO in the lung (indicated with arrowhead) (200× magnification). Data are expressed as mean ± SD (*n* = 6 per group). ***p < 0.001, compared with the control group; ^++^ *p* < 0.01, ^+++^ *p* < 0.001, compared with the IR group

Compared with the control group, the I/R group had significantly increased malondialdehyde levels, carbonyl contents, and numbers of MPO-positive cells in the lung tissue (*p* < 0.05, Fig. 5a–c). Treatment with FK866 significantly mitigated these increases.

### Effect of FK866 on lung pathology

**Fig. 6:**
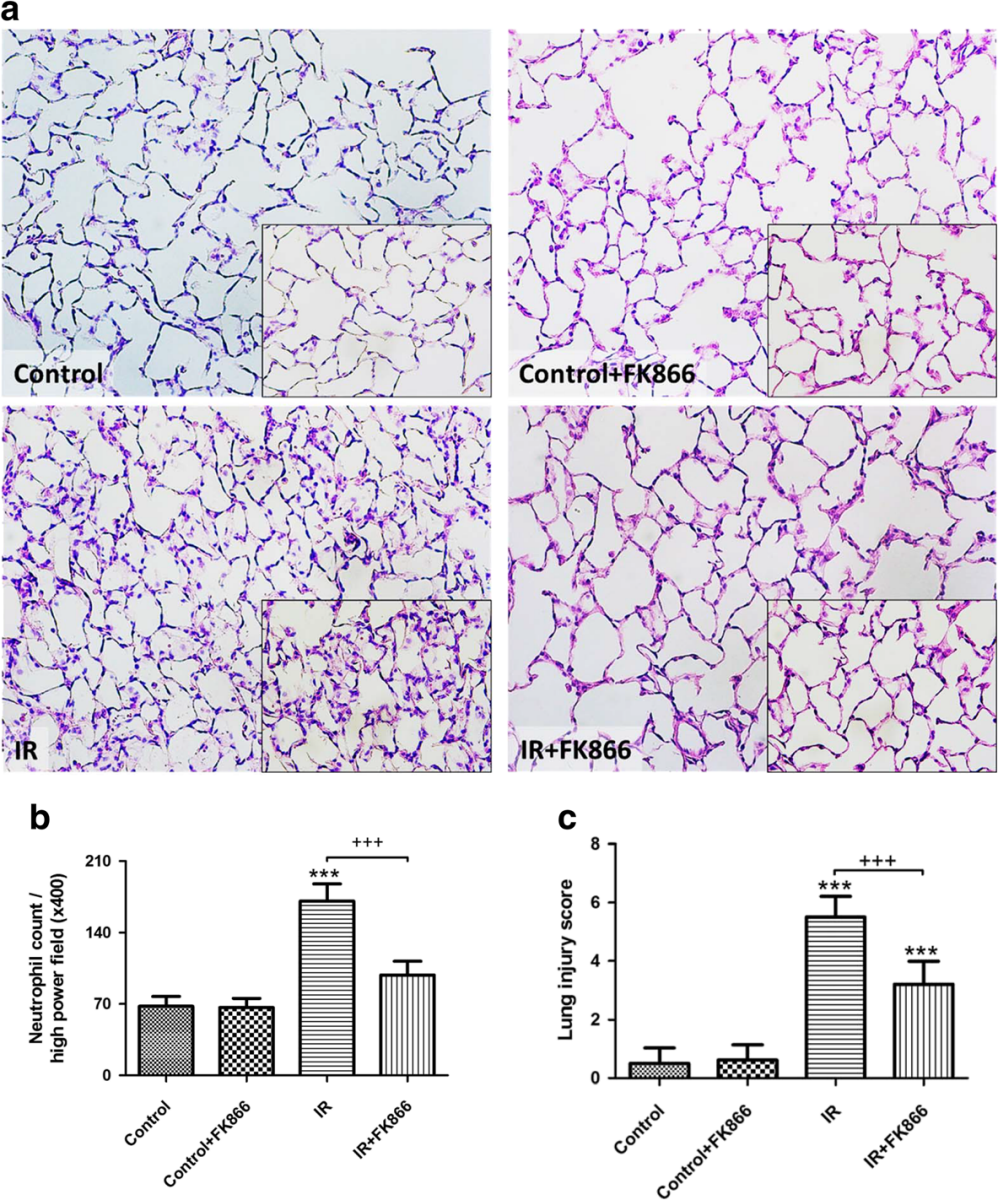
Effect of FK866 on lung pathology. As shown by a representative micrograph of lung tissue (400× magnification) (**a**), neutrophil infiltration and septal edema were increased in the ischemia-reperfusion (IR) group. FK866 treatment significantly attenuated these histopathological changes, the numbers of neutrophils per high power field (400× magnification) (**b**), and the lung injury scores (**c**). Data are expressed as mean ± SD (*n* = 6 per group). ***p < 0.001, compared with the control group; ^+++^ *p* < 0.001, compared with the IR group

The histological studies disclosed distinct morphological evidence of lung injury, including thickening of the alveolar walls characterized by interstitial edema and leukocyte infiltrates in the I/R group compared with the control group (Fig. 6a). FK866 treatment significantly reduced histological changes, neutrophil infiltration (Fig. 6b), and lung injury scores (Fig. 6c) in the I/R group.

### Effects of FK866 on Bcl-2 and caspase-3 protein expression in lung tissue

**Fig. 7:**
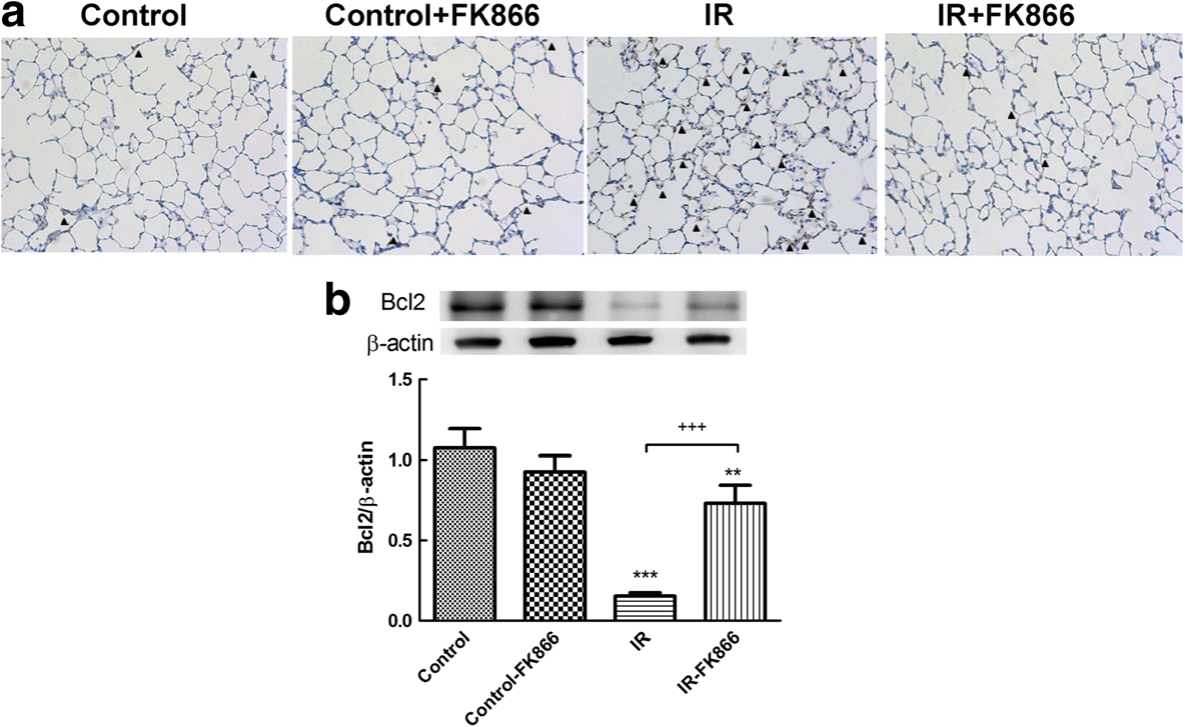
Effect of FK866 on the expression of caspase-3 and Bcl-2 in lung tissue. **a** Immunohistochemistry for active caspase-3 in the lung (*indicated with an arrowhead*) (200× magnification). **b** Western blot analysis of Bcl-2 protein in the lung tissue. β-actin served as a loading control for cytoplasmic proteins. Representative blots are shown. Ischemia-reperfusion (IR) significantly decreased Bcl-2 protein expression and induced caspase-3 activation in the lung tissue. FK866 treatment significantly increased Bcl-2 protein expression and attenuated the signals for active caspase-3 in the IR group. Representative blots are shown. Data are expressed as mean ± SD (*n* = 6 per group). ***p < 0.01, compared with the control group; ^+++^ *p* < 0.01, compared with the IR group

The intensity of activated caspase-3-immunolabelled cells was significantly greater in the I/R group than in the control group. FK866 treatment significantly decreased the number of caspase-3 immunolabeled cells (Fig. 7a). Bcl-2 protein content in lung tissue was substantially reduced in the I/R groups compared with the control group. FK866 treatment significantly mitigated the decline of Bcl-2 levels in the lung tissue (Fig. 7b).

### Effect of FK866 on the mitogen-activated protein kinase (MAPK) signaling pathway and MKP-1 induction in lung tissue

**Fig. 8:**
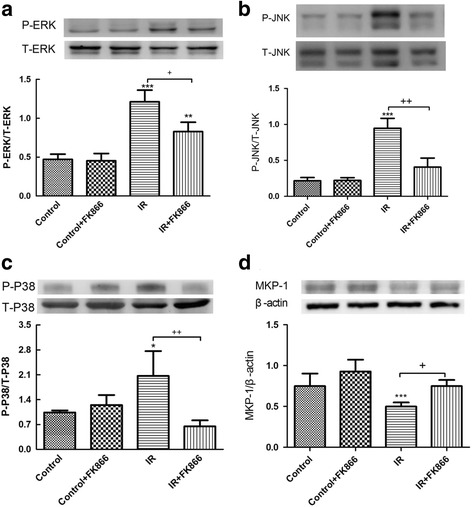
Effect of FK866 on MAPK and MKP-1 expressions in lung tissue. The phosphorylation of ERK (**a**), JNK (**b**), and p38 (**c**) was enhanced in the ischemia-reperfusion (IR) group. FK866 treatment attenuated these effects. In contrast, the expression of MKP-1 protein (**d**) was decreased in the IR group but reversed by FK866 treatment. β-actin served as the loading control. A representative blot is shown. All data are shown as mean ± SD (*n* = 6 per group). *p < 0.05, ***p < 0.001, compared with the control group; ^+^ *p* < 0.05, ^++^ *p* < 0.01, compared with the IR group

I/R significantly increased activation of the mitogen-activated protein kinase (MAPK) pathway, including ERK, JNK, and p38 phosphorylation in lung tissue. All three MAPKs activated by I/R were reduced by the administration of FK866 (Fig. 8a–c). In contrast, the MKP-1 protein level in lung tissue was significantly lower in the I/R groups than in the control groups but was significantly enhanced with FK866 treatment (Fig. 8d).

### Effect of FK866 on the NF-κB signaling pathway

**Fig. 9:**
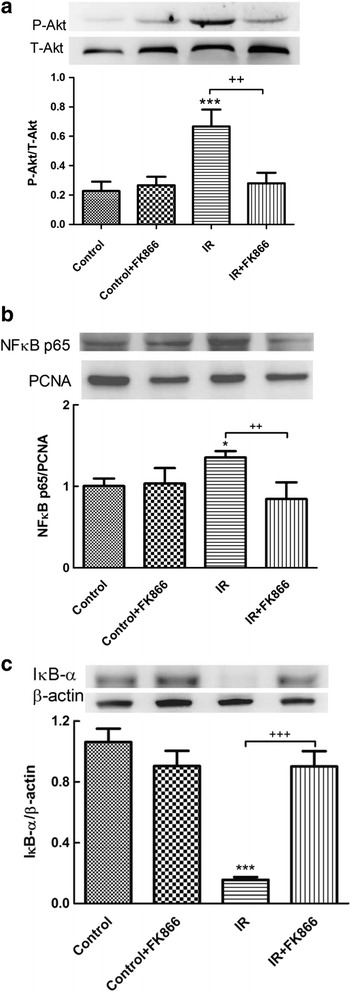
Effect of FK866 on NF*-*κB activation and Akt phosphorylation in lung tissues. FK866 reduced Akt phosphorylation (**a**) and nuclear NF-κB p65 levels (**b**), and increased IκB-α levels (**c**) in ischemia-reperfusion (IR)-induced lung injury. PCNA and β-actin served as loading controls for nuclear and cytoplasmic proteins, respectively. Representative blots are shown. Data are expressed as mean ± SD (*n* = 6 per group). *p < 0.05, ***p < 0.001, compared with the control group; ^++^ *p* < 0.01, ^+++^ *p* < 0.001, compared with the IR group

The levels of NF-κB p65 in the nucleolus and Akt phosphorylation were significantly increased (Fig. 9a, c), whereas the level of IκB-α in the cytoplasm was significantly decreased in the I/R group compared with the control group (Fig. 9b). FK866 treatment significantly increased IκB-α levels, and attenuated NF-κB p65 levels and Akt phosphorylation.

### Effect of FK866 in A549 epithelial cells subjected to H/R

**Fig. 10:**
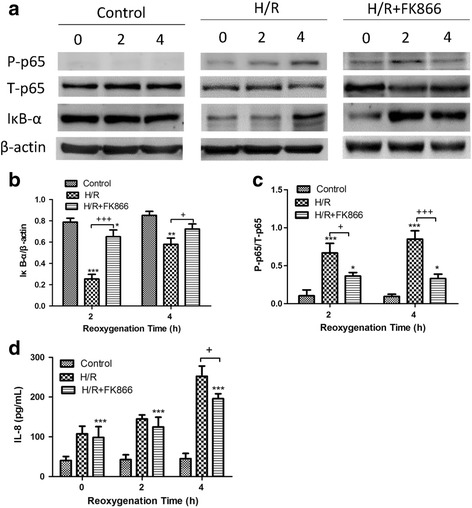
Effect of FK866 on A549 cells subjected to hypoxia-reoxygenation (H/R). **a** A representative Western blot of NF-κB nuclear translocation in the lung tissue. β-actin served as the loading control. FK866 significantly reduced the increase of degradation of IκB-α (**b**), phosphorylated NF-κB p65 (**c**) at 2 h and 4 h, and IL-8 production (**d**) at 4 h in A549 cells exposed to H/R. Data are expressed as mean ± SD (*n* = 6). *p < 0.05, **p < 0.01, ***p < 0.001 compared with the control group. ^+^ *p* < 0.05, ^+++^ *p* < 0.001, compared with the H/R group

FK866 significantly reduced the increase of phospho-NF-κB p65 and the decrease of IκB-α at 2 h and 4 h after H/R in A549 cells (Fig. 10a-c). Furthermore, FK-866 significantly reduced the levels of IL-8 at 4 h in the H/R group (Fig. 10d).

## Discussion

The results of our study demonstrated that FK866, an inhibitor of NAMPT enzymatic function, significantly ameliorated multiple indices of I/R-induced acute lung injury, including vascular barrier dysfunction, PAP, pulmonary neutrophil influx, BALF TNF-α, CINC-1, and IL-6 production, oxidative stress, apoptosis, and tissue damage compared with control animals. In addition, FK866 inhibited I/R-induced MAPK and Akt activation, IκB-α degradation, and nuclear translocation of NF-κB. Moreover, FK866 treatment had a similar beneficial effect on A549 epithelial cells subjected to H/R, corroborating the observations in the rat lung tissues. This indicates that FK-866 exerts its protective effects through multiple signaling cascades. Our experiments revealed the importance of NAMPT in the pathophysiology of I/R-induced lung injury.

After I/R injury, there was a significant increase in the NAMPT protein expression in the lung tissue, similar to the results of previous studies showing that NAMPT protein was highly upregulated in VILI and intestinal ischemia-reperfusion [7, 11]. This could be due to infiltration of activated inflammatory cells with upregulated NAMPT expression into injured lung tissue [9]. Upregulation of NAMPT expression is also observed in cytokine, LPS, mechanical stress–challenged human lung endothelial cells and human alveolar epithelial cells, and LPS-stimulated RAW264.7 cells [7, 11, 18]. These results strongly support a significant role for NAMPT in the inflammatory events observed in ALI/ARDS. Moreover, we showed that NAMPT inhibition by FK-866 attenuated I/R-induced increases in NAMPT protein. This attenuation may block various I/R-associated inflammatory responses, thereby leading to a significant reduction of lung damage. This finding is consistent with a previous study showing that FK866 decreased the degree of positive staining of NAMPT in the spinal cord of mice subjected to spinal cord injury [19].

Vast evidence has demonstrated that oxidative stress has a significant contribution in the pathogenesis of ALI/ARDS [20]. In addition, neutrophil-derived oxygen radicals disrupt endothelial barrier function and integrity, and increase plasma leakage and lung tissue edema. Our data demonstrated that FK-866 suppressed oxidative stress as reflected by attenuating the protein carbonylation and peroxidation of membrane lipids in I/R lung tissue. In addition, FK-866 attenuated I/R-evoked increased neutrophil infiltration in the lung tissue, as evidenced by diminishing numbers of neutrophils and MPO-positive cells. This attenuation blocks the interaction between neutrophils and the endothelium, and reduces the production of proinflammatory cytokines and free radicals by activated neutrophils. Therefore, the anti-oxidative and anti-inflammatory effects of FK-866 appeared to attenuate lung edema as shown by the lower W/D and LW/BW ratios, reduced K_f_, and decreased protein concentration in the BALF. These results agreed with those of other investigations showing that NAMPT inhibitors have the ability to attenuate vascular permeability and neutrophil infiltration in VILI, intestinal I/R, and LPS-induced lung injuries [10, 11].

Previous investigations have implicated a complex network of inflammatory cytokines and chemokines in mediating, amplifying, and perpetuating the lung injury process [12]. Our experiment showed that FK-866 significantly attenuated the increased levels of inflammatory mediators such as proinflammatory TNF-α, CINC-1 and IL-6 in the BALF after I/R-induced lung injury. Our findings were also comparable with those in previous investigations showing that FK866 alleviated TNF-α production, thereby reducing inflammation, and preventing the I/R lung injury [11, 19]. In parallel with these results, FK-866 significantly suppressed caspase-3 activation and increased anti-apoptotic expression of Bcl-2 after I/R lung injury. The observations were also similar to those seen in intestinal I/R induced lung injury and spinal cord injury [11, 19]. FK866 has been employed as an antitumor agent through NAD and subsequent ATP depletion, resulting in apoptosis in many malignant cell lines [21]. TNF-*α*, one of the major inflammatory mediators in ALI/ARDS, can initiate the apoptotic cascade through the death receptor/caspase pathway [22]. Because FK866 inhibits TNF-*α* production, it is reasonable to speculate that FK866 inhibits apoptosis in I/R-induced ALI, at least partly, through an indirect pathway [11]. Furthermore, this discrepancy suggests that FK866 has different effector mechanisms for apoptosis of tumor cells and suppression of inflammatory reactions in inflammatory cells [4].

NF-kB is a master regulator of inflammatory responses because it activates the transcription of a cascade of proinflammatory cytokines and chemokines. The activity of NF-κB is regulated primarily by the IκB family of inhibitory proteins, which are conjugated with NF-κB in the cytoplasm [23]. Inappropriate activation of NF-κB is implicated in the pathogenesis of ALI/ARDS. Akt also participates in signaling pathways that lead to NF-κB activation and increased NF-κB-dependent transcription [24]. Moreover, Akt-dependent events contribute to the development and perpetuation of ALI [25]. Our prior investigations revealed that I/R-induced lung damage caused IκB degradation and NF-κB activation [15, 26]. Hong et al. demonstrated that *NAMPT* ^+/−^ mice exhibited a dramatic attenuation of the VILI-mediated NF-κB pathway in the lungs [9]. In the present experiment, FK866 suppressed the activation of the NF-κB signaling pathway in the rat lungs exposed to I/R by inhibiting Akt phosphorylation, degradation of IκBα and nuclear translocation of NF-κB. The inhibition of NF-κB activity resulted in decreased production of proinflammatory cytokines such as TNF-α, CINC-1, and IL-6. Furthermore, we conducted in vitro cell culture studies by using the A549 epithelial cell line to elucidate the direct effects of FK866 on alveolar epithelial cells. In line with the findings in the rat lungs, FK866 significantly inhibited IκBα degradation and, consequently, NF-κB p65 phosphorylation, and the production of IL-8 in A549 cells exposed to H/R. This finding was also consistent with an investigation by Matsuda et al. demonstrating that FK866 inhibited NF-κB activation in mouse lungs subjected to intestinal I/R, and LPS-stimulated RAW264.7 cells [11]. However, in an experimental compression model of spinal cord injury, FK866 treatment prevented the activation of NF-κB but not IκBα degradation [19]. Therefore, the precise molecular mechanisms by which NAMPT inhibition exerts its effect in the NF-κB signaling pathway need clarification.

The activation of MAPKs such as p38, ERK, and JNK is implicated in the inflammatory process of ALI/ARDS. The inhibition of p38, ERK, and JNK MAPK, effectively diminishes LPS and peritonitis-induced lung inflammation [27–29]. The MAPK signaling pathways are regulated by opposing regulatory repressors from MKP-1 [30]. MKP-1 knockout mice had increased inflammatory responses with higher levels of inflammatory mediators and more episodes of multiple organ failure after LPS challenge [31]. In our previous study, I/R induced phosphorylation of p38, ERK, and JNK; it also decreased the level of MKP-1 protein in lung tissue [17]. In contrast, FK-866 treatment activated MKP-1 expression and interrupted I/R-induced activation of MAPK. This may consequently restrain widespread inflammation in I/R-induced lung injury. Extracellular NAMPT is reported to trigger p38, ERK, and JNK phosphorylation and stimulates diverse biological processes in various types of cells [1, 32]. However, whether intracellular NAMPT triggers the same intracellular pathways remains unknown.

The function of NAMPT in various diseases is not completely recognized. One reason for this lack of clarity could be the inability to discriminate between the intracellular and extracellular actions of NAMPT. NAMPT is enzymatically active both intracellularly and extracellularly [32]. Extracellular NAMPT may also act in a non-enzymatic way to regulate activation of inflammatory cells by increasing surface expression of costimulatory molecules and inducing IL-1β, IL-6, and TNF-α production through a currently unidentified membrane receptor [32]. The fundamental pathological event of ischemic stroke is the loss of blood supply and subsequent oxygen/nutrition shortage, which are similar in key pathophysiological processes to I/R lung injury. Accumulating evidence from in vitro and in vivo experiments reveals that NAMPT provides cerebral protection in ischemic stroke [3]. NAMPT inhibition exacerbated brain infarction in a rat model of ischemic stroke, whereas local NAMPT overexpression in the brain and NAMPT enzymatic action protected against ischemia-induced cerebral strokes [3]. Therefore, further investigations are needed to explore these contradictory findings.

## Conclusions

Collectively, we demonstrated that FK866, a NAMPT inhibitor attenuated lung I/R injury by decreasing lung edema, production of inflammatory cytokines, reactive oxygen species, apoptosis, and NFκB and MAPK signaling. The protective actions of FK866 in this study displayed a pleiotropic manner. Therefore, the pharmacological inhibition of NAMPT might serve as an effective approach for the treatment of I/R-induced lung injury. A better understanding of its physiological action is a prerequisite for the use of NAMPT inhibitors such as FK866 as a therapeutic option in these inflammatory diseases.

## Abbreviations

ALI: Acute lung injury
AnxA1: Annexin A1
ARDS: Acute respiratory distress syndrome
BALF: Bronchoalveolar lavage fluid
Bcl-2: B-cell lymphoma-2
CINC-1: Cytokine-induced neutrophil chemoattractant-1
ERK: Extracellular signal-related protein kinase
H/R: Hypoxia-reoxygenation
I/R: Ischemia/reperfusion
IκB-α: Inhibitor of NF-κB-α
JNK: c-Jun N-terminal kinase
K_f_: Vascular filtration coefficient
LPS: Lipopolysaccharide
LW/BW: Lung weight to body weight ratio
MKP-1: Mitogen activated protein kinase phoshphotases-1
MPO: Myeloperoxidase
NAD: Nicotinamide adenine dinucleotide
NAMPT: Nicotinamide phosphoribosyltransferase
p38: p38 protein kinase
PAP: Pulmonary arterial pressure
PVP: Pulmonary venous pressure
VILI: Ventilator-induced lung injury
W/D: Wet weight to dry weight ratio
